# Horizontal Dispersal Limitation and Vertical Environmental Filtering Drive Ciliate Community Assembly in a Tibetan Plateau Deep Lake

**DOI:** 10.3390/microorganisms14020422

**Published:** 2026-02-11

**Authors:** Chen Wang, Ruizhi An, Yang Liu

**Affiliations:** Key Laboratory of Biodiversity and Environment on the Qinghai-Tibet Plateau, Ministry of Education, School of Ecology and Environment, Xizang University, Lhasa 850000, China

**Keywords:** ciliate, community assembly, Qinghai–Xizang plateau, environmental threshold, Basomtso Lake

## Abstract

The Qinghai–Xizang Plateau, known as the “Asian Water Tower”, hosts numerous lakes that are highly sensitive to climate change. Ciliates, key microbial eukaryotes in aquatic ecosystems, play crucial roles in biogeochemical cycling and food web dynamics. However, their community assembly mechanisms in such extreme habitats remain poorly understood. In July 2020, we investigated the ciliate community in Basomtso Lake. A total of 15 sampling sites were established along the horizontal gradient, and 11 vertical depth samples were collected at a central site (B15), resulting in 75 water samples for eDNA analysis. Using 18S rRNA gene high-throughput sequencing, we identified 610 ciliate amplicon sequence variants (ASVs), with the class Spirotrichea being the dominant taxonomic group. Distance–decay relationships indicated a significantly stronger community turnover rate along the vertical gradient compared to the horizontal gradient. Analyses using the neutral community model and null model revealed that community assembly was primarily stochastic. However, increasing vertical environmental heterogeneity enhanced the role of deterministic, niche-based selection. Random forest modeling identified resistivity (RES) and water temperature (WT) as the key predictors for horizontal and vertical community variation, respectively. Furthermore, Threshold Indicator Taxa Analysis (TITAN) detected specific taxa exhibiting pronounced sensitivity to gradients in RES and WT. Our findings demonstrate that horizontal community structure is governed primarily by dispersal limitation, whereas vertical zonation is shaped by environmental filtering driven primarily by RES and WT gradients under extreme plateau conditions. This study provides new insights into the mechanisms sustaining microbial diversity and ecosystem resilience in climatically vulnerable high-altitude lakes.

## 1. Introduction

Protozoa, as an important group of eukaryotic microorganisms, play a key role in the material cycling and energy flow of aquatic ecosystems [1,2]. Among them, ciliates are a group characterized by complex morphological structures and highly differentiated functions [3]. They regulate microbial community composition and abundance by preying on bacteria, algae, and other small protozoa. Simultaneously, they serve as high-quality prey for zooplankton and fish, thereby forming a crucial link between the microbial loop and the classical food web [4,5]. Furthermore, through their metabolic and cellular activities, ciliates promote organic matter decomposition and nutrient regeneration, influencing the biogeochemical cycles of carbon, nitrogen, and phosphorus [6,7]. Due to their high sensitivity to water quality changes, ciliate community structure is frequently used as a bioindicator for aquatic ecosystem health, holding significant value for ecological assessment and environmental monitoring [8,9].

The Qinghai–Xizang Plateau, renowned as the “Asian Water Tower”, sustains the largest and most extensive assemblage of alpine lakes in China, accounting for over 50% of the nation’s total lake area [10]. Glacial meltwater and precipitation converge here, forming a scattered distribution of saline and freshwater lakes. These alpine lakes not only play a crucial role in regulating regional climate but also serve as the headwaters of major Asian rivers such as the Yangtze, Yellow, and Lancang rivers [11]. Furthermore, the alpine lake systems influence the climate of East Asia and even the globe through water vapor circulation, and their ecosystems are highly sensitive to climate change due to their inherent fragility [12,13]. Globally, alpine lakes have emerged as critical sentinels for studying climate change impacts on aquatic ecosystems, with recent research revealing significant long-term shifts in ciliate communities across European high-mountain lakes, likely driven by rising temperatures and enhanced UV radiation [14]. Additionally, thermal tolerance studies of freshwater ciliates indicate that climate warming will progressively shift planktonic communities from algivorous to bacterivorous species, fundamentally altering microbial food web structures in high-altitude lakes [15]. Located in southeastern Tibet, Basomtso is a large glacial barrier lake situated in the broad valley of the Ba River, the largest tributary of the Nyang River. As a typical representative of alpine lakes, it plays a significant ecological role in maintaining regional climate stability and protecting biodiversity. Particularly important is that Basomtso experiences minimal anthropogenic disturbance, largely preserves its primitive ecosystem, and responds rapidly and sensitively to climate change. These characteristics make it an ideal natural laboratory for studying the structure of aquatic biological communities and their environmental responses under natural conditions, thus attracting widespread attention from scholars both domestically and internationally [16]. Currently, biological research on Basomtso has primarily focused on groups such as bacteria [17], fungi [18], algae [19], and fish [20], whereas ciliate communities in this extreme alpine lake habitat remain relatively understudied.

Utilizing 18S rRNA gene high-throughput sequencing, this study was conducted to systematically characterize the ciliate communities in Basomtso Lake, with the objectives of revealing their composition, deciphering their assembly mechanisms, and identifying the environmental drivers along both horizontal and vertical gradients. Here, “horizontal” refers to surface spatial variation among the 15 sampling sites, while “vertical” denotes depth-related changes from surface to deep water (109.5 m) at the central site. The research provides essential biodiversity data and scientific insights into the maintenance of microbial communities in extreme plateau environments. It also contributes to the development of biological indicators for monitoring climate change effects on plateau lake ecosystems, offering significant scientific value for safeguarding aquatic biological resources and ensuring ecological security on the Qinghai–Xizang Plateau.

## 2. Materials and Methods

### 2.1. Study Area and Sampling

**Figure 1:**
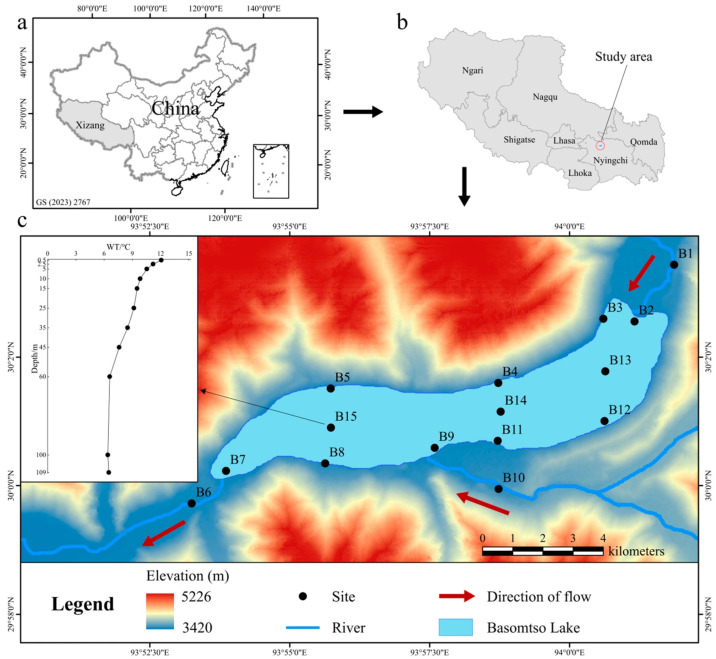
Sampling site distribution map in Basomtso Lake. (**a**) Location of the study area in Xizang, China. (**b**) Specific cities of the Basomtso Lake in Xizang. (**c**) Horizontal and vertical distribution of the sampling sites in the Basomtso Lake.

The Basomtso Lake, also known as Cuogao Lake (29°59′–30°04′ N, 93°53′–94°01′ E), is in the Nyang River basin within Gongbo’gyamda County, Nyingchi City, Xizang Autonomous Region [17] (Figure 1, Table S1). It is approximately 18 km in length, with an elongated, crescent-shaped morphology divided into eastern and western basins. The lake surface area is about 27 km^2^, primarily recharged by precipitation and glacial meltwater. Surrounded by mountains, the lake region experiences a mild, plateau temperate semi-humid monsoon climate, with an average annual precipitation of 646 mm concentrated mainly from April to October. The maximum water depth reaches 120 m, and the lake surface lies at an average elevation of 3480 m [19].

In July 2020, we conducted a comprehensive sampling campaign in Basongco Lake to investigate its aquatic microbial communities. Based on the lake’s ecological characteristics, functional zoning, and environmental gradients, fifteen sampling sites were established at the surface layer of rivers and the lake. These sites were further classified into three functional zones according to geographical location and habitat type: Water samples were collected from the surface layer (0.5 m depth) of rivers and the lake using a handheld water sampler. The central site (B15) was selected for detailed vertical profiling, with a total water depth of 109.5 m measured using a traditional sounding weight. Guided by vertical gradients in water temperature and depth, water samples were collected from 11 discrete depth layers, corresponding to three vertical strata: the Epilimnion, Metalimnion, and Hypolimnion (Table S2). For vertical sampling, water from depths shallower than 40 m was collected using a plexiglass water sampler, while samples from depths exceeding 40 m were obtained with a closing-capped deep water sampler (QCC15, a Niskin-type sampler). For each site, three independent replicates were collected/analyzed to ensure statistical robustness. A total of 75 water samples were collected from 25 sampling sites (three replicates per site) for subsequent environmental DNA (eDNA) analysis. The central site (B15) at 0.5 m depth was assigned three replicates, which were used in both the horizontal and vertical sample groups.

At each sampling point (including both horizontal sites and vertical layers), three independent 5 L composite samples were collected as field replicates. Each 5 L sample was first pre-filtered through a 200 μm mesh to remove coarse particles. The filtrate was then concentrated by vacuum filtration through a 0.22 μm pore-size polycarbonate membrane (Millipore). His step was repeated four times (yielding four membranes per replicate), with 350 mL of filtrate processed per membrane. All membranes containing microbial biomass were immediately flash-frozen in liquid nitrogen and stored at −80 °C until DNA extraction.

### 2.2. Physical and Chemical Variables

Water temperature (WT, °C) and resistivity (RES, Ω·cm) were measured with a HI98192 microprocessor-based resistivity meter (HANNA, Villafranca Padovana, Italy). The pH, electrical conductivity (EC, μS/cm), salinity (salt, mg/L), and total dissolved solids (TDS, mg/L) were determined using a PCSTestr35 waterproof multi-parameter tester (EU-TECH, Sacramento, CA, USA). Dissolved oxygen (DO, mg/L) was measured with a HI98193 portable dissolved oxygen meter (HANNA, Italy). Turbidity (TUR, NTU) was assessed using a HI98703 multi-parameter turbidity meter (HANNA, Italy), and ammonia nitrogen (NH_3_-N, mg/L) was quantified with a HI83399 compact photometer (HANNA, Italy).

### 2.3. DNA Extraction and High-Throughput Sequencing

Environmental DNA was extracted using the PowerSoil DNA Isolation Kit (MoBio Laboratories, Carlsbad, CA, USA). DNA quality was assessed by 1% agarose gel electrophoresis, and its concentration and purity were determined with a NanoDrop 2000 spectrophotometer (Thermo Fisher Scientific, Wilmington, DE, USA). The V9 hypervariable region of the eukaryotic 18S rRNA gene was amplified by polymerase chain reaction (PCR) using the primers 1391f (5′-GTACACACCGCCCGTC-3′) and 1510r (5′-TGATCCTTCTGCAGGTTCACCTAC-3′). Amplicon sequencing was performed on the Illumina NovaSeq 6000 platform (2 × 250 bp paired-end) by Novogene (Beijing, China).

### 2.4. Bioinformatics Analyses

Raw sequencing reads were first processed with Cutadapt (v1.9.1) to remove barcode and primer sequences, generating quality-controlled raw reads [21]. Subsequent steps were performed in QIIME2 [22,23]. The DADA2 plugin was applied for filtering, merging paired-end reads, and removing chimeras and low-quality sequences [24]. By performing 100% similarity clustering (i.e., exact deduplication), DADA2 resolved amplicon sequence variants (ASVs) with higher resolution and accuracy. Each deduplicated sequence was referred to as an ASV, and their abundance profile constitutes the ASV table. Taxonomic assignment of ASVs was conducted against the native BIOM database (v5.0.0) [25]. Only ASVs classified as ciliates were retained for downstream analyses.

### 2.5. Statistical Analyses

Data analysis and plotting were performed using R language (version 4.4.2) and Origin 2024. Data processing and parameter settings were performed using the “itol.toolkit” package (version1.1.12) [26], and the iTOL web server [27] was subsequently employed for data visualization and phylogenetic tree construction. Geographical distances were calculated using the “geosphere” package (version 1.5-20) [28], and environmental distances were calculated using the “stats” package (R version 4.4.2) [29], to quantify phylogenetic community structure across spatial and environmental gradients, and to test for phylogenetic conservatism in environmental tolerances. Blomberg’s *K* value was calculated using the “picante” package (version 1.8.2) [30]. *K* value describes the phylogenetic conservation of traits; a *K* value close to 0 indicates a convergent or random evolution pattern, while a *K* value greater than 1 indicates a deeper phylogenetic signal and ecological niche conservation [31]. The Fritz & Purvis *D* test for discrete data was evaluated using the “caper” package (version 1.0.3) to assess the significant phylogenetic signal of the data [32]. The *D* value was converted to −*D* + 1 to indicate whether the evolution of each environmental variable did not show a significant phylogenetic signal (−*D* + 1 = 0) or was more conservative than expected by chance (−*D* + 1 > 0) [33]. Species-average relative abundance, occurrence frequency, and predicted occurrence frequency in the neutral model were calculated using the “Hmisc” (version 5.2-5), “minpack.lm” (version 1.2-4), and “stats4” (R version 4.4.2) packages [34]; the phylogenetic zero model was calculated using the “iCAMP” package (version 1.5.12) [35]; to evaluate the species co-occurrence pattern, the “EcoSimR” package (version 0.1.0) was used to calculate the checkerboard score; positive SES values indicate separated species co-occurrence patterns; negative SES values indicate aggregated species co-occurrence patterns; a close SES value, or when the empirical C-score is not significantly different from the zero distribution, indicates a random species co-occurrence pattern [36]. Random forest analysis was performed using the “randomForest” package (version 4.7-1.2) [37]. BioEnv was used to obtain the optimal environmental factor combination [38,39], and the Mantel test and graphical visualization were performed using the “phyloseq” (version 1.50.0), “ggplot2” (version 4.0.0), and “linkET” (version 0.0.7.4) packages [40]. Environmental threshold analysis was performed using the “TITAN2” package (version 2.4.3) [41]. The sample distribution map was drawn using ArcGIS 10.8.

## 3. Results

### 3.1. Composition of Horizontal and Vertical Ciliate Communities

**Figure 2:**
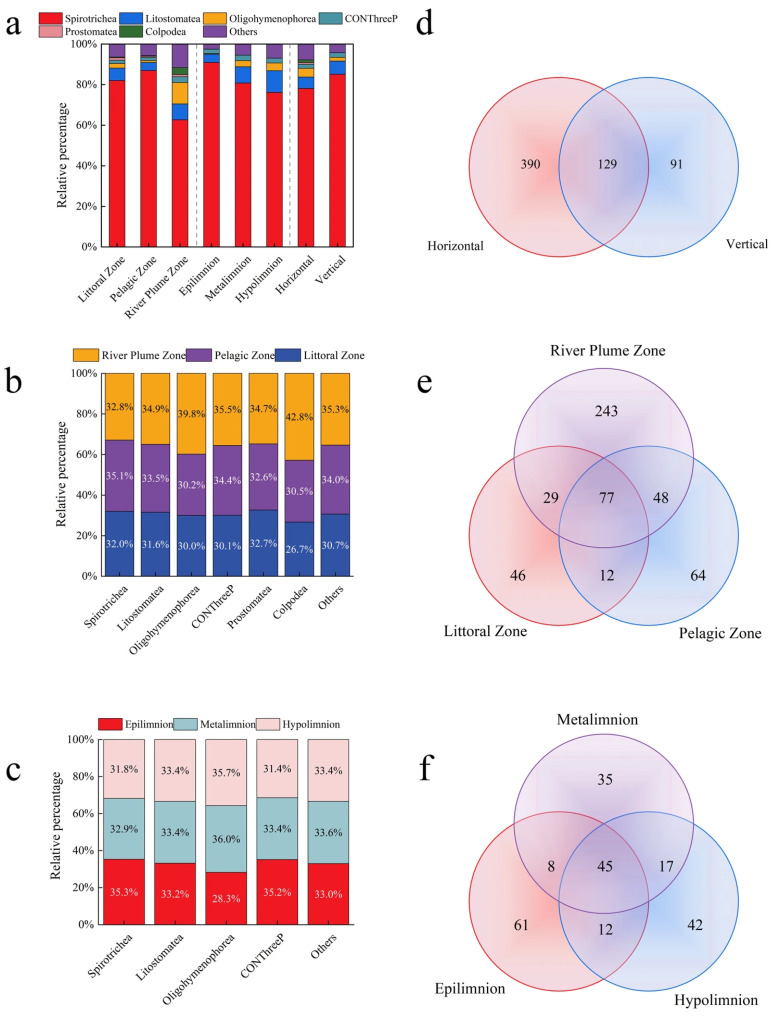
Composition of the ciliate community in Basomtso Lake. (**a**) Taxonomic composition across different lake regions. (**b**) Relative contribution of distinct horizontal communities. (**c**) Relative contribution of distinct vertical communities. (**d**) Between surface and deep water communities. (**e**) Unique and common ASVs in different regions. (**f**) Unique and common ASVs among ciliate communities at various depths.

**Figure 3:**
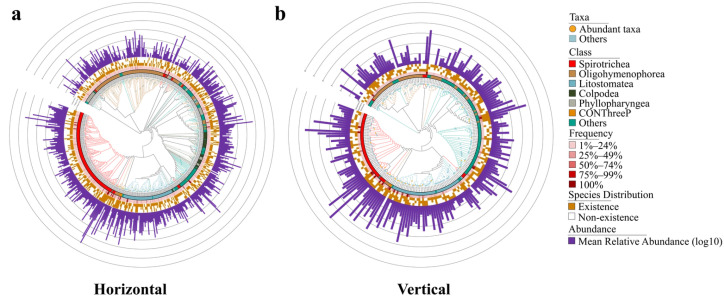
Phylogenetic community structure of ciliates across directions in Basomtso Lake. (**a**) Structure of the horizontal ciliate zonation. (**b**) Structure of the vertical ciliate zonation.

The ciliate community in Basomtso Lake yielded a total of 610 amplicon sequence variants (ASVs). In the horizontal samples, a total of 519 ASVs were obtained, with the dominant classes (relative abundance greater than 1%) being Spirotrichea, Litostomatea, Oligohymenophorea, CONThreeP, Colpodea, and Phyllopharyngea, accounting for 78.11%, 5.63%, 4.31%, 2.02%, 1.33%, and 1.01% respectively (Figure 2a,b). A total of 53 ASVs (relative abundance > 0.1%) were considered dominant (Figure 3a), with ASV1064 showing the highest relative abundance (55.97%) (Table S3). In the vertical samples, a total of 220 ASVs were obtained, with the dominant classes (relative abundance greater than 1%) being Spirotrichea, Litostomatea, CONThreeP, and Oligohymenophorea, accounting for 85.07%, 6.54%, 2.28%, and 1.83% respectively (Figure 2a,c). Among 30 dominant ASVs (relative abundance > 0.1%) (Figure 3b), ASV1064 remained the most abundant (46.77%) (Table S3). In total, 129 ASVs were shared between the horizontal and vertical dimensions, while 390 ASVs were exclusively horizontal and 91 ASVs were exclusively vertical (Figure 2d).

In the horizontal ciliate communities, there were significant differences in the composition between in the River Plume Zone, Littoral Zone, and Pelagic Zone, with the Spirotrichea class remaining the dominant group (Figure 2a). The River Plume Zone had 397 ASVs of ciliate, the Littoral Zone had 164 ASVs, and the Pelagic Zone had 201 ASVs. There were a total of 77 ASVs of ciliate in the three regions (Figure 2e). In the vertical communities, there were significant differences in the composition between in Epilimnion, Metalimnion, and Hypolimnion (Figure 2a). The Spirotrichea class remained the dominant group. The Epilimnion region had 126 ASVs of ciliate, the Metalimnion region had 105 ASVs, and the Hypolimnion region had 116 ASVs. A total of 45 ciliate ASVs were shared among the three regions (Figure 2f).

### 3.2. Analysis of the Attenuation of Ciliate Communities in the Horizontal and Vertical Directions

**Figure 4:**
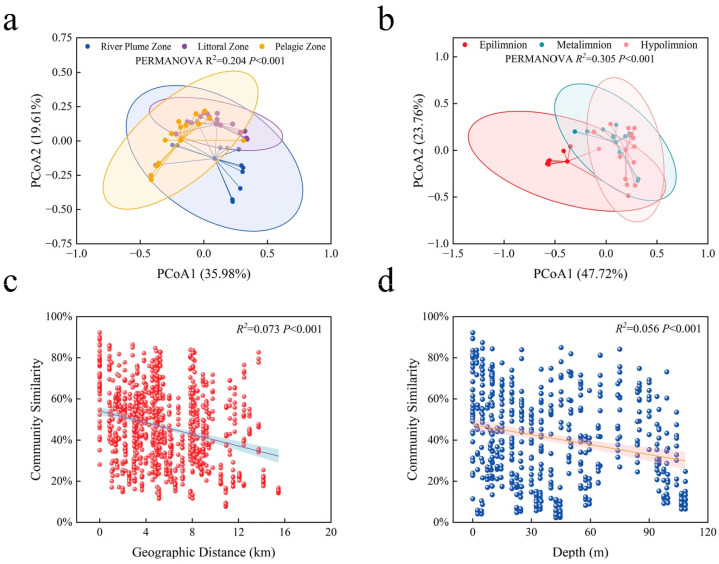
Spatial patterns in ciliate community structure in Basomtso Lake. (**a**) PCoA of horizontal communities. (**b**) PCoA of vertical communities. (**c**) Distance–decay relationship across geographical distance. (**d**) Distance–decay relationship across water depth.

A principal coordinates analysis (PCoA) was conducted to examine both horizontal and vertical distribution patterns of ciliate communities in Basomtso Lake. Horizontal analysis revealed that the first two principal coordinates explained 55.59% of the total community variation (Axis 1: 35.98%; Axis 2: 19.61%), with PERMANOVA confirming significant differences among ciliate communities across different regions (*p* < 0.001) (Figure 4a). Vertical analysis demonstrated even stronger explanatory power, with the first two axes accounting for 71.48% of the variation (Axis 1: 47.72%; Axis 2: 23.76%); PERMANOVA again indicated significant regional differentiation (*p* < 0.001) (Figure 4b).

Distance–decay relationships were further evaluated for both spatial dimensions. Geographic distance exhibited a significant negative correlation with community similarity across horizontal gradients (Figure 4c), indicating that ciliate community composition becomes increasingly dissimilar with increasing spatial separation. Similarly, water depth showed a significant negative relationship with community similarity in the vertical profile (Figure 4d), demonstrating depth-driven community stratification.

### 3.3. Systematic Evolutionary Signals of Ciliate Communities in Horizontal and Vertical Directions

**Figure 5:**
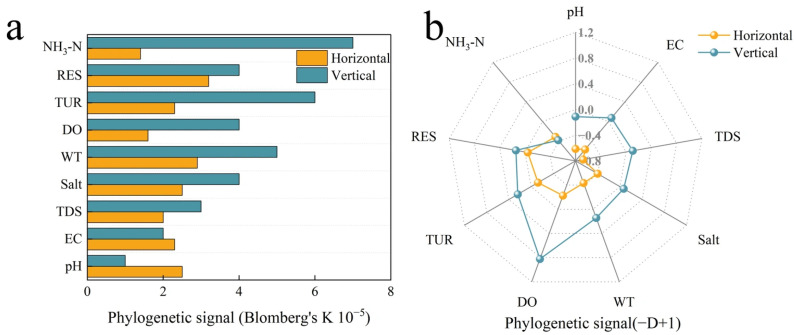
Phylogenetic signal in traits of the Basomtso Lake ciliate community. (**a**) Signal assessed by Blomberg’s *K*. (**b**) Signal assessed by the Fritz & Purvis’s D statistic.

In the horizontal ciliate community, Blomberg’s *K* test revealed that the three environmental variables (Figure 5a), DO, TDS, and NH_3_-N, exhibited the most significant phylogenetic signal. EC and TUR showed marginal significant phylogenetic signals, while other environmental and spatial variables did not display significant phylogenetic signals (*p* > 0.05). The results of Fritz & Purvis’s *D* test presented a completely different pattern (Figure 5b). The −*D* + 1 values of all environmental factors were negative, and the *p* values were all greater than 0.59, indicating that, when these continuous environmental factors were binary processed, their phylogenetic conservatism completely disappeared. This significant difference between the *K* test and *D* test results precisely reflects the sensitivity differences in the detection of phylogenetic signals between continuous traits and binary traits.

The phylogenetic signal pattern of the vertical ciliate community shows distinct characteristics from that of the horizontal community. The *K* test results indicate that only NH_3_-N shows a significant phylogenetic signal (Figure 5a), while TDS and salt exhibit marginal significant features. It is particularly noteworthy that the *Z* values of pH and depth are even negative, suggesting that the phylogenetic signals of these factors in the vertical dimension are weaker than the random expectation. The *D* test results show that the DO value is the highest (0.84) (Figure 5b), and the −D + 1 values of factors such as TUR, WT, and RES are also greater than 0, but the *p* values of these results are all greater than 0.14, suggesting only a weak conservative trend. The D test result of NH_3_-N is opposite to that of the *K* test result, once again highlighting the differences in the sensitivity of different testing methods to data types. Overall, the phylogenetic signal intensity of environmental factors in the vertical community is significantly weaker than that in the horizontal community, and this phenomenon is likely related to the environmental homogenization effect caused by vertical mixing in the lake.

DO exhibited the clearest phylogenetic signal in both the horizontal dimension’s *K* test and the vertical dimension’s *D* test, indicating that it has a crucial influence on the assembly of the ciliate community. Secondly, the spatial variables did not show significant phylogenetic signals in either dimension, suggesting that the direct impact of pure spatial processes on the phylogenetic structure of ciliate may be relatively limited.

### 3.4. Selection of Environmental Factors in Horizontal and Vertical Directions

**Figure 6:**
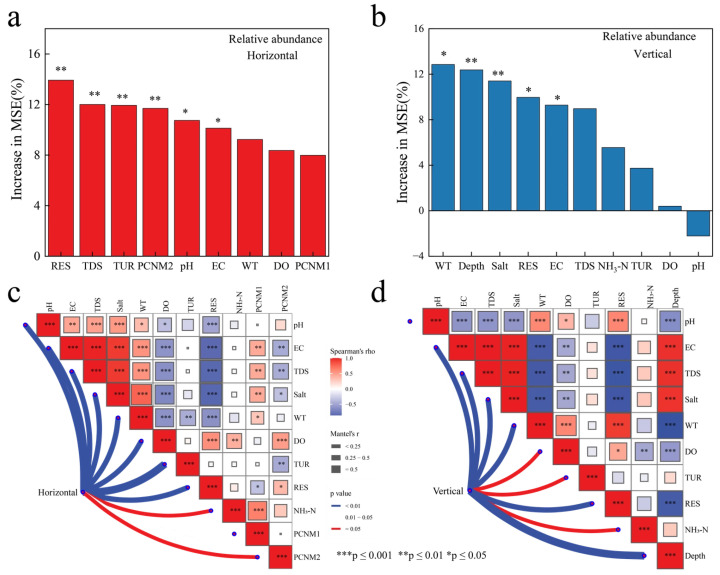
Identification of key environmental factors shaping ciliate communities in Basomtso Lake. (**a**) Random forest analysis for the horizontal community. (**b**) Random forest analysis for the vertical community. (**c**) Mantel test assessing environment–community correlations for the horizontal community. (**d**) Mantel test assessing environment–community correlations for the vertical community.

To select the optimal environmental factors in both the horizontal and vertical directions of Basomtso. random forest importance values based on relative abundance were used. RES was the strongest predictor for the horizontal community of ciliate (Figure 6a), and WT was the strongest predictor for the vertical community of Basomtso ciliate (Figure 6b). Further analysis using BIOENV was conducted to determine the best combination of environmental factors influencing the ciliate communities. The best environmental factor combination affecting the horizontal community of ciliate was WT, TUR, and PCNM1 (the first principal coordinate of neighbor matrices, representing spatial autocorrelation), with the highest explanatory power of 0.592 (Table S4). The best environmental factor combination affecting the vertical community of ciliate was depth, with the highest explanatory power of 0.524 (Table S4). To further clarify the effects of different environmental factors on the ciliate communities, the Mantel test analysis was used. The horizontal community was influenced by the combined effects of RES and spatial factors (Figure 6c, Table S5), while the vertical community was influenced by WT, DO, and RES (Figure 6d, Table S6).

### 3.5. Horizontal and Vertical Community Assembly

**Figure 7:**
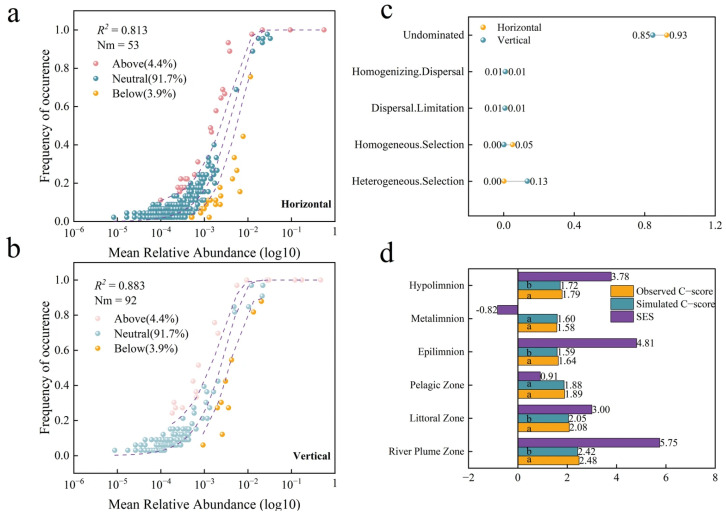
Community assembly of ciliates in Basomtso Lake. (**a**) Neutral model for the horizontal community. (**b**) Neutral model for the vertical community. (**c**) Null model. (**d**) Checkerboard score (C-score). Same letters above bars indicate no significant difference; different letters indicate significant difference (*p* < 0.05).

Based on the neutral model, a systematic analysis was conducted on the community assembly process of the Basomtso ciliate community in both the horizontal and vertical directions. It was found that both spatial dimensions exhibited significant neutral characteristics. In the horizontal direction, the fitting results of R^2^ = 0.813 and Nm = 53 indicated that the neutral process played a dominant role in the community assembly (Figure 7a). This suggests that the dispersal ability of the ciliate community in this area is strong, with frequent individual exchanges between populations, which helps maintain a high level of species diversity. It indicates that environmental heterogeneity has a relatively small impact on the community structure in this direction, and the competition among species and ecological niche differences are not significant. This pattern may be related to the relatively uniform water mixing and relatively stable physical and chemical conditions, thereby weakening the role of environmental selection, allowing random processes to dominate in community assembly. In the vertical direction, the fitting results of *R^2^* = 0.883 and *Nm* = 92 indicated that the neutral process played an important role in the vertical community construction (Figure 7b). The results demonstrate pronounced vertical population connectivity, with dynamic individual turnover preserving diverse ciliate communities. This pattern may be related to the strong vertical mixing of the water body and the small size of ciliate individuals that are easily carried by water currents, thereby weakening the role of environmental selection and allowing the neutral process to still dominate in community assembly. It may also be that ciliate has strong connectivity, with frequent migrations of individuals between different water layers, further promoting the random distribution of species.

Based on the null model, it was found that the random process dominated the construction of the horizontal ciliate community, while the ecological selection effect was weak, and the environmental selection effect was significantly weakened. Specifically, the proportion of Homogenizing Dispersal was only 1.21%, while the proportion of Undominated was as high as 92.5%, indicating that the spatial distribution of the vast majority of species could not be explained by deterministic ecological processes (such as selection or dispersal limitation), which is in line with the prediction of the neutral theory. The proportion of Heterogeneous Selection was only 0.2%, the proportion of Homogeneous Selection was 5.05%, and the proportion of Dispersal Limitation was 1.01% (Figure 7c). These extremely low values collectively indicate that the environmental heterogeneity in the horizontal direction is extremely low, the water body mixing is intense, the passive dispersal of ciliate is frequent, the differences in species competition cannot be amplified, and ecological differentiation almost does not exist. “Undominated” accounted for an absolute dominance, meaning that the community structure was mainly driven by neutral processes such as random birth, death, and dispersal, which was highly consistent with the results of the previous neutral model. Compared with the horizontal direction, the ecological selection effect in the vertical direction was significantly enhanced, but the random process still dominated. The proportion of Heterogeneous Selection reached 13.45%, much higher than 0.2% in the horizontal direction, indicating that the environmental gradient in the vertical direction had a significant selection effect on the community structure. Some species were selectively retained or excluded due to adaptive differences. However, “Undominated” still reached 84.66%, indicating that, although environmental selection existed, the distribution of most species could not be explained by deterministic processes, and neutral processes such as random dispersal and drift were still the main controlling mechanisms for the vertical community assembly. All three were in the low value range, which may further indicate that, although there is environmental heterogeneity in the vertical direction, ciliate, with their high-dispersal ability and the vertical mixing of the water body, effectively weakened the effects of dispersal limitation and homogeneous dispersal, allowing random processes to still occupy an absolute advantage.

Further, the zero model of the chessboard score was used to determine the species co-occurrence patterns and the relative importance of deterministic and stochastic processes in community formation. In the horizontal direction, the species co-occurrence patterns of the River Plume Zone and the Littoral Zone significantly deviated from the random expectations (*p* < 0.05), indicating the presence of strong non-random ecological processes. Among them, the standardized effect value (*SES* = 5.75) of the River Plume Zone was the highest, and the species co-occurrence showed a significant aggregation, possibly due to factors such as environmental filtering or mutualism; the non-randomness of the Littoral Zone was slightly weaker (*SES* = 3.00), but still exhibited a significant species aggregation pattern. In contrast, the observed values of the Pelagic Zone were close to the simulated mean (*SES* = 0.91) (Figure 7d), indicating that its species co-occurrence was mainly dominated by stochastic processes. In the vertical direction, the species co-occurrence patterns of different water layers showed significant differences. The observed value of the Epilimnion (1.64) was significantly higher than the simulated mean (1.59), with an SES of 4.81 (*p* < 0.05) (Figure 7d), showing a strong non-random aggregation pattern, and environmental filtering or positive interactions may dominate the species coexistence in this layer. The Hypolimnion also showed significant non-randomness, but the effect value was lower than that of the Epilimnion, possibly reflecting weaker environmental selection pressure, while the observed value of the Metalimnion was close to the simulated mean, conforming to the random co-occurrence pattern, indicating that this layer may be dominated by neutral processes. These results reveal the spatial heterogeneity of ecological process and neutral process in the vertical stratification of the water body. The biological interactions or environmental filtering effects in the Epilimnion and Hypolimnion are stronger, while the community construction in the Metalimnion may be more random. Overall, the construction of the ciliate community in Basomtso is dominated by neutral processes, but the environmental filtering effect strengthens in the vertical direction, and local areas exhibit non-random co-occurrence patterns. This pattern is consistent with the theory of community assembly of highly diffusive organisms in homogeneous environments, and reflects the influence of water physical stratification and micro-environment heterogeneity.

### 3.6. Horizontal and Vertical Direction Environmental Thresholds

**Figure 8:**
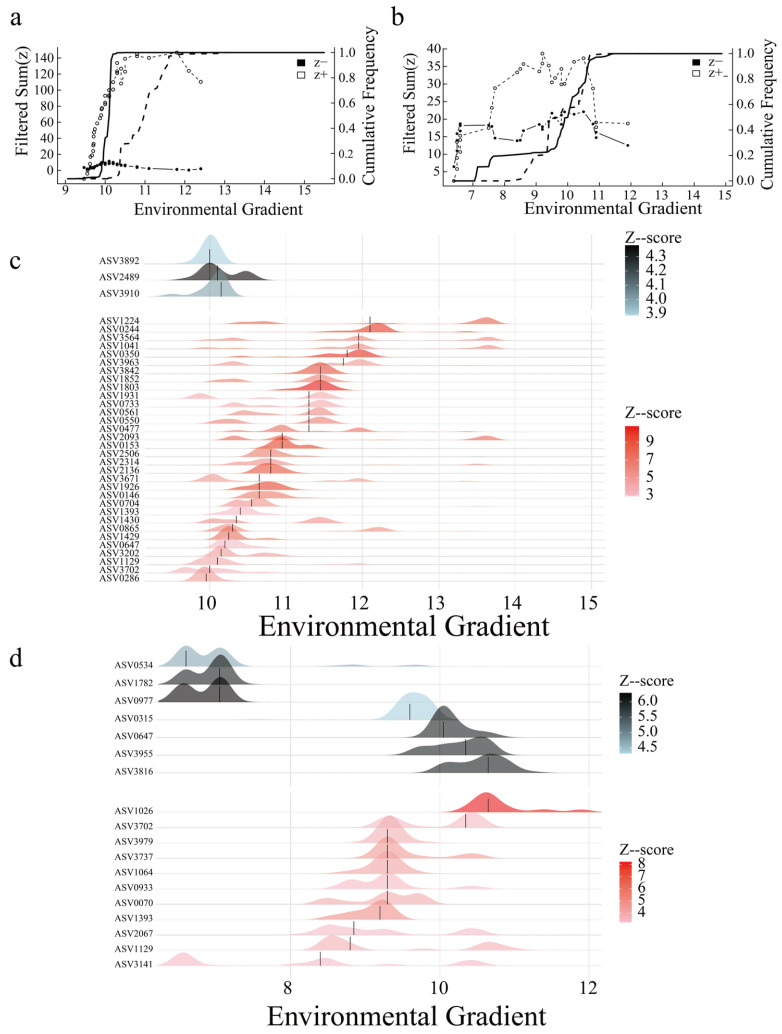
Environmental thresholds for ciliate communities in Basomtso Lake. (**a**) Response patterns of horizontal community groups to RES. (**b**) Significant indicator groups for the horizontal community identified by RES. (**c**) Response patterns of vertical community groups to WT. (**d**) Significant indicator groups for the vertical community identified by WT. Statistical notes: sum(z−) represents the total response of decreasing indicator groups (solid black symbols); sum(z+) represents that of increasing indicator groups (unfilled symbols).

In this study, random forest was used to screen the key environmental factors in the horizontal direction as RES, and the key environmental factors in the vertical direction as WT. Based on this, TITAN threshold analysis was adopted, combined with the response characteristics of the ciliate communities under different environmental gradients. The results showed that the threshold for the decrease in the horizontal ciliate community was 9.65 μS·cm^−1^, and the threshold for the increase was 11.65 μS·cm^−1^. A total of 168 significantly responding ASVs were found, including 120 positive responding species and 48 negative responding species (Figure 8a). Among them, ASV1064 (*IndVal* = 83.8), ASV3141 (*IndVal* = 81.62), etc. can be used as reliable biomarkers for the decrease in the horizontal ciliate community, and ASV3529 (*IndVal* = 100), ASV1224 (*IndVal* = 96.31) (Figure 8c, Table S7), etc. can be used as reliable biomarkers for the increase in the horizontal ciliate community. The threshold for the decrease in the vertical ciliate community was 6.6 °C, and the threshold for the increase was 10.65 °C. A total of 74 significantly responding ASVs were found, including 30 positive responding species and 44 negative responding species (Figure 8b). Among them, ASV3955 (*IndVal* = 94.92), ASV3816 (*IndVal* = 91.2), etc. can be used as reliable biomarkers for the decrease in the vertical ciliate community, and ASV1064 (*IndVal* = 89.2), ASV2067 (*IndVal* = 87.26), etc. can be used as reliable biomarkers for the increase in the vertical ciliate community (Figure 8d, Table S8).

## 4. Discussion

### 4.1. Horizontal and Vertical Distribution Patterns of Ciliate

This study utilized environmental DNA technology to detect 610 ciliate ASVs in Basomtso, which was more than the 273 OTUs (Operational Taxonomic Units) found in Weishan Lake survey [42] and the 312 OTUs extracted directly from the Yellow Sea cold water mass. However, it was lower than the 451 OTUs obtained by the DNA elution method in the latter study [43]. This cross-study difference was mainly constrained by technical factors, with different denoising methods and clustering thresholds affecting the classification of ASVs/OTUs; there was a deviation in the classification resolution between PR2 and SILVA and other reference databases; the sensitivity of universal primers for detecting various ciliate groups was different [44,45,46]. The dominant ciliate classes detected in Basomtso were the Spiralia and Spumaria classes, and this community structure was highly consistent with the high-altitude meadow soil ecosystem, confirming the typical characteristics of the ciliate fauna in extreme high-altitude environments [47]. It indicates that the cold and high-altitude environment may have shaped a community pattern dominated by the Spiralia class through selection pressures such as low temperature, strong ultraviolet radiation, and nutrient deficiency [14]. This may be related to the characteristics of the Spiralia class, such as its well-developed ciliary apparatus and high feeding efficiency—this group can efficiently ingest microplankton and organic debris through its well-developed membranous mouthparts [48]. Compared to high-altitude water bodies, the ciliate communities in inland water bodies and soil systems exhibit more complex group composition and environmental gradient responses. The higher organic matter reserves, more stable water conditions, and resource supply in the soil environment enhance the resource competition process and support the relative increase in opportunistic groups (such as the oligochaete class) in the community [49,50]. Existing studies have shown that different environmental gradients can promote the relative increase in the proportion of groups with specific functional adaptations, and the unique cell mouth structure of the Spumaria class gives it an advantage in ingesting attached bacteria and organic particles [51,52].

In the horizontal direction, the high number of ASVs (397) in the River Plume Zone far exceeds that in the Littoral Zone (164) and the Pelagic Zone (201). This is mainly attributed to the microhabitat diversity formed by the input of terrestrial nutrients and the enrichment of suspended matter in the estuarine area, which provides abundant ecological niches and food resources for ciliate. The low diversity in the Littoral Zone may be limited by water level fluctuations and substrate stability, while the Pelagic Zone reflects the selective pressure of homogeneous open water resources [53]. In the vertical distribution, the slight dominance of Epilimnion (126 ASVs) may be due to the primary productivity driven by light availability [54]. However, the small differences between the three water layers and the relatively high proportion of shared species (45 ASVs) indicate that this water body may have strong vertical mixing or turbidity-mediated light inhibition, weakening the shaping effect of the light gradient on the community in typical stratified lakes. In the deep water body, the physical stratification process may have a more significant regulatory effect on the community structure of ciliate compared to other environmental gradients [55,56].

This study utilized PCoA and PERMANOVA analyses to reveal significant differentiation of the Basomtso ciliate communities in both horizontal and vertical dimensions, and this differentiation had a high statistical explanatory rate. The total explanatory rate of the first two axes of the PCoA for the horizontal community was 55.77%, with axis one contributing more (34.79%), indicating that the differences in community structure among different sampling points were mainly driven by geographical distance (dispersal limitation) or local environmental heterogeneity, while the explanatory rate for the vertical community was higher (71.48%), suggesting that the water depth gradient had a much stronger impact on the ciliate community than the horizontal direction. The significance of the distance attenuation and depth attenuation effects further supported this conclusion. In the horizontal direction, community similarity decreased with the increase in geographical distance, which is in line with the classic “distance–decay” theory, indicating that the cumulative effect of dispersal limitation or local environmental differences plays a key role in shaping the community structure. In the vertical direction, the trend of community similarity decreasing with water depth was more obvious, which is consistent with the strong environmental stratification in deep water lakes [57]. Compared to inland water bodies, the ciliate communities in Basomtso exhibited stronger spatial differentiation. Inland low-altitude lakes usually have shallow water bodies and sufficient wind mixing, resulting in a weak vertical environmental gradient, and the PCoA explanatory rate may be lower. Typical inland deep water lakes have a seasonal mixing, anthropogenic eutrophication, and enhanced watershed connectivity, which homogenize the species pool and weaken or even reverse the distance attenuation effect [58]. In contrast, as a high-altitude deep water lake, Basomto’s physical enclosure, low human interference, and strong environmental gradient jointly enhance the ecological selection effect of spatial factors, making the community differentiation more easily observable [59].

### 4.2. Phylogeny of Ciliates

This study reveals the unique ecological processes of the plateau lakes through the dual test of phylogenetic signals: at the horizontal scale, DO and NH_3_-N drive the differentiation of lineages, while at the vertical scale, the mixing effect leads to the weakening of conservatism. At the horizontal scale, DO, TDS, and NH_3_-N show significant phylogenetic signals (Blomberg’s *K*), indicating that the adaptability of ciliate to key water quality parameters is lineage-dependent; DO and nitrogen nutrients, especially, are the core factors driving the construction of the microbial community. The *K* value of DO in this study may differ significantly from that of low-altitude lakes, possibly reflecting the stronger environmental variability of plateau lakes that weakens the selection pressure, and also coinciding with the theory that ecological niche conservatism is regulated by environmental heterogeneity. This theory suggests that species tend to retain ancestral ecological niche characteristics during evolution, and the intensity of environmental heterogeneity directly regulates the degree of this conservatism. Specifically, in habitats with high environmental heterogeneity, strong environmental selection pressure drives ecological differentiation among closely related species to avoid competition, thereby weakening the detection of phylogenetic signals [60,61]; conversely, in relatively homogeneous and stable habitats, due to the lack of strong selection pressure, species are more likely to retain ancestral environmental adaptation characteristics, resulting in enhanced phylogenetic clustering and more significant phylogenetic signals [35]. At the vertical dimension, only NH_3_-N maintains a significant phylogenetic signal, while pH and depth even show negative signals (Z < 0), indicating that vertical mixing significantly weakens the environmental filtering effect. DO shows the clearest phylogenetic signal in both the horizontal dimension’s *K* test and the vertical dimension’s D test (Fritz & Purvis’s D), strongly supporting the core functional deep conservatism hypothesis [62,63]. This hypothesis suggests that basic metabolic functions such as respiration have deep conservatism on the phylogenetic tree, and DO, as the core limiting factor of aerobic respiration, has strong phylogenetic signal that reflects the conservative adaptation strategy of the ciliate group to the oxidative environment over the long evolutionary process. The spatial variables show no significant signals in both dimensions, contrasting sharply with the formation of bacterial biogeography in the South China Sea due to human activities [64]. This difference may result from the relatively original environmental characteristics of Qinghai–Xizang Plateau lakes—the evolutionary selection effect of natural geographical gradients is much weaker than the anthropogenic eutrophication gradient, indicating that spatial processes in inland highly disturbed water bodies may indirectly affect the phylogenetic structure through environmental synergy. The vertical environmental gradient of low-altitude lakes is relatively gentle, and the microbial community usually shows stronger vertical stratification conservatism [65]. NH_3_-N is the only factor showing signals in both the horizontal and vertical communities; studies have shown that nitrogen metabolic functions show differentiated conservatism at different phylogenetic depths, with the basic nitrogen cycling function being widely conservative and specific processes being restricted to specific branches [66]. The dual signals of NH_3_-N in this study may stem from the conservation dependence of ciliate symbiotic bacteria or its own metabolic ability on NH_3_-N, which is also reflected in the research on microbial communities in urban rivers—the ammonia nitrogen gradient in eutrophic rivers significantly screens out specific β-Deinococcus lineages [67].

### 4.3. Ciliate Community Assembly

The comprehensive analysis based on the neutral model and the null model indicates that the ciliates community of Basomtso exhibits significant neutral characteristics in both the horizontal and vertical dimensions. However, the environmental selection effect in the vertical direction is significantly stronger than that in the horizontal direction. This spatial heterogeneity reflects the dynamic trade-off between random processes and deterministic processes at the microscale. This reflects the typical strong vertical mixing of the plateau lake. This mixing is mainly driven by large diurnal temperature differences, continuous wind-driven convection, and the injection of meltwater cold water, thereby disrupting the stable thermal stratification [68]. This may be due to the seasonal thermocline blocking the vertical migration of ciliate organisms. The plateau lakes exhibit enhanced vertical circulation due to climate and topographic forcing, which is conducive to the homogenization of microbial communities, rather than the common stratified isolation phenomenon in other regions [69]. The oligotrophic state of Basomtso and the physical mixing of the water body maintain more efficient vertical connectivity [68]. The zero model analysis further validates the absolute dominance of the neutral process. In the horizontal direction, the proportion of Undominated is 92.5%, while Homogenizing Dispersal is only 1.21%, indicating the low environmental heterogeneity and strong dispersal capacity of the plateau environment [70]. Studies have shown that the continuous water environment of natural lakes is more dominated by dispersal than the fragmented river network [71]. In the vertical direction, although Undominated still accounts for 84.66%, the proportion of Heterogeneous Selection has risen to 13.45%. However, this selective signal is far stronger than that in the horizontal direction, indicating that the vertical environmental gradient is exerting a weak but detectable selection effect. This selective intensity is much lower than that of inland lakes, possibly due to the deeper water layer of Basomtso still maintaining higher dissolved oxygen and low temperature stability, avoiding the common bottom anoxia stress in inland eutrophic lakes, thereby reducing the intensity of environmental filtering [70]. The spatial heterogeneity revealed by the zero model score provides key evidence for understanding the scale dependence of process intensity. In the horizontal direction, the River Plume Zone and the Littoral Zone show significant non-random co-occurrence, while the Pelagic Zone conforms to the random expectation (SES = 0.91). This habitat differentiation pattern corresponds to the “source–sink” theory [72]. The River Plume Zone forms a unique “ecological island” due to the suspended particles and dissolved organic carbon carried by glacial meltwater, promoting the aggregation of specific ciliate organisms through density constraint priority effects or environmental filtering; the Littoral Zone is influenced by terrestrial inputs and the micro-environment of aquatic plants roots, and the species interaction network becomes more complex. In contrast, the Pelagic Zone has a high degree of environmental homogeneity, conforming to the typical random dispersal pattern of “core community” [73]. In the vertical direction, the non-randomness of Epilimnion and Hypolimnion contrasts with the random pattern of Metalimnion. The possible mechanism of Basomtso lies in the fact that Epilimnion is affected by UV radiation and wind stirring, and ciliate organisms adapted to high light intensity and temperature fluctuations achieve non-random coexistence through positive interactions; Hypolimnion, due to the degradation of settled organic matter, generates a microscale anaerobic micro-environment, and groups preferring low oxygen and anaerobic symbiosis are selectively retained. The random coexistence of Metalimnion implies that, although there is a density gradient, ciliate organisms can still effectively cross through with their small size and strong swimming ability, avoiding dispersal limitations [74]. This is consistent with the “microbial neutrality paradox” theory—that the high-dispersal ability of microorganisms leads to a random distribution at the local scale, but weak selection signals remain at the large-scale environmental gradient [75]. Compared to the eutrophic lakes in the eastern plains, the oligotrophic state of Basomtso significantly weakened the intensity of resource competition, reducing the relative fitness differences among species and highlighting the neutral drift effect. This study confirmed that, in conditions of weak dispersal limitation and low environmental stress, neutral processes can dominate up to 90% of the community variation, supporting the “randomity gradient theory”, that is, the importance of randomness increases as the organism size decreases and environmental heterogeneity decreases. Moreover, the weak existence of selection signals in the vertical direction indicates that, even in high-dispersal systems, weak environmental filtering can still be detected by highly sensitive zero models [76].

### 4.4. Key Driving Factors of Ciliate Communities

This study determined that RES and WT were the strongest predictors of the horizontal and vertical communities, respectively, using the random forest model. In the study of the Great Lakes region of North America, water temperature was generally considered to be the core driving factor for vertical stratification, while the horizontal-distributed communities were more influenced by nutrient gradient or suspended matter concentration [77]. The best predictor combination for the horizontal community of Basomtso is (WT, TUR, PCNM1), in which PCNM1 (a spatial variable) indicates the unique topographic limiting effect of plateau lakes. This difference may be due to the low temperature, strong ultraviolet radiation, and low nutrient salt environment of the lakes in the Qinghai–Xizang Plateau, resulting in the biological community being more sensitive to physical parameters than chemical parameters. This may also be because the strong environmental selection pressure of the plateau lakes weakened the species pool effect, causing the relative contribution rate of spatial processes to increase [78]. Basomtso, as a deep water lake, may be due to the narrow coastal zone and rich large aquatic plants, or it may be related to the spatial heterogeneity of suspended particles and the redistribution of endogenous nutrient salts [16]. Consistent with the “resource patch hypothesis”, the horizontal directional resource redistribution caused by wind-driven hydrodynamic processes is the key driver of the microbial biogeographic pattern [79]. Vertically, the ciliate community of Basomtso is only significantly affected by water depth, which may be related to its shorter water mixing cycle and low productivity, which is consistent with the typical characteristics of high-altitude lakes. It may also be due to its unique double-temperature thermocline structure [19]. Additionally, the WT thresholds of 6.6 °C and 10.65 °C determined by TITAN exactly correspond to the typical temperature range of the thermocline in Qinghai–Xizang Plateau lakes. When the bottom water temperature is too low, the phosphorus release rate of the sediment significantly decreases, leading to a decrease in the abundance of bottom ciliate [74,80]. The ciliate community stratification is mainly regulated by the vertical distribution of DO and Chl-a, and the thermocline effect is relatively weak [81]. Studies have revealed that ciliate form abundance peaks in the upper part of the thermocline, synchronizing with the vertical migration of microzooplankton, constituting a key link of the “nutrient pump” [82]. ASV1064 of Baosong Co is enriched in the Epilimnion with WT > 10.65 °C, possibly participating in similar vertical carbon output regulation, but its low threshold of <6.6 °C for the Hypolimnion indicates the presence of the indicator species ASV0534, suggesting that the ciliate in the Qinghai–Xizang Plateau may have evolved stronger cold-adaptation capabilities [83].

This study provides important insights into ciliate community assembly in Basomtso Lake. Future research would benefit from increased sampling frequency to capture seasonal and inter-annual dynamics, particularly valuable given the extreme of the Qinghai–Xizang Plateau. Second, integrating eDNA metabarcoding with complementary approaches, such as traditional microscopy and single-cell sequencing, would provide deeper functional and physiological context for our molecular findings. Third, establishing long-term monitoring programs would facilitate tracking the responses of these sensitive ecosystems to ongoing climate change. Finally, experimental manipulations of key environmental gradients (e.g., temperature, resistivity) would enable quantification of the relative contributions of stochastic and deterministic processes under future warming scenarios. Together, these extensions would build upon our current framework to advance our understanding of microbial diversity maintenance and ecosystem resilience in the “Asian Water Tower.”

## 5. Conclusions

This study clarifies the spatial patterns and assembly mechanisms of ciliate communities in the deep, high-altitude Basongco Lake. Spirotrichea dominated the community composition as the absolute dominant group, exhibiting significant spatial differentiation along both horizontal and vertical gradients, with stronger vertical differentiation. Neutral processes dominate community assembly, but vertical environmental heterogeneity enhances ecological selection. RES and WT are key drivers of horizontal and vertical communities, respectively, and sensitive indicator taxa for relevant environmental thresholds were identified. These findings offer significant contributions to microbial ecology in extreme environments. By quantifying the shifting balance between stochastic and deterministic assembly processes along critical environmental gradients, this research provides a mechanistic framework for understanding biodiversity patterns in deep plateau lakes. The identification of RES and WT as primary drivers, along with their associated bioindicators, establishes a critical baseline for monitoring ecological responses to climate change in these sensitive “Asian Water Tower” ecosystems. This study underscores the vulnerability of vertically structured microbial communities to environmental change and highlights the necessity of incorporating both horizontal and vertical dimensions in conservation strategies for alpine aquatic biodiversity.

## Data Availability

The data presented in this study are available on request from the corresponding author. The data are not publicly available due to privacy restrictions.

## Supplementary Materials

The following supporting information can be downloaded at: https://www.mdpi.com/article/10.3390/microorganisms14020422/s1, Table S1: Geographic location and environmental characteristics of the horizontal sampling sites in Basomtso Lake; Table S2: Depths and environmental characteristics of the vertical sampling profile at site B15 in Basomtso Lake; Table S3: Relative abundances of the top 10 dominant ciliate ASVs in horizontal and vertical communities; Table S4: Results of the BIOENV analysis linking environmental factors to ciliate communities in Basomtso Lake; Table S5: Mantel test results for correlations between environmental variables and the horizontal ciliate community in Basomtso Lake; Table S6: Mantel test results for correlations between environmental variables and the vertical ciliate community in Basomtso Lake; Table S7: Indicator taxa and change points for the horizontal ciliate community identified by Threshold Indicator Taxa Analysis (TITAN) along the resistivity (RES) gradient; Table S8: Indicator taxa and change points for the vertical ciliate community identified by Threshold Indicator Taxa Analysis (TITAN) along the water temperature (WT) gradient.

## References

[1] Zhu L., Feng L., Zhang D., Shi F., Zou X., Yang Q., He S., Zhu W. (2025). Eukaryotic plankton community and assembly processes in a large-scale water diversion project in China. Sci. Rep..

[2] Vickerman K. (1992). The diversity and ecological significance of Protozoa. Biodivers. Conserv..

[3] Gao F., Warren A., Zhang Q., Gong J., Miao M., Sun P., Xu D., Huang J., Yi Z., Song W. (2016). The all-data-based evolutionary hypothesis of ciliated protists with a revised classification of the phylum Ciliophora (Eukaryota, Alveolata). Sci. Rep..

[4] Asghar U., Mukherjee I., Sonntag B., de Paula C.C.P., Kasalický V., Bulzu P.-A., Singh A.P., Shabarova T., Piwosz K., Šimek K. (2025). Morphological and molecular analyses of season-specific responses of freshwater ciliate communities to top-down and bottom-up experimental manipulations. mSystems.

[5] Romano F., Pitta P. (2021). Relationships of pelagic ciliate with the microbial food web components at a coastal station in the oligotrophic Eastern Mediterranean Sea: Temporal and vertical variability. J. Plankton Res..

[6] Goldman J.C., Caron D.A., Andersen O.K., Dennett M.R. (1985). Nutrient cycling in a microflagellate food chain: I. Nitrogen dynamics. Mar. Ecol. Prog. Ser..

[7] López-Abbate M.C., Garzón-Cardona J.E., Silva R., Molinero J.-C., Ruiz-Etcheverry L.A., Martínez A.M., Gilabert A.S., Lara R.J. (2025). The bacteria–protist link as a main route of dissolved organic matter across contrasting productivity areas on the Patagonian Shelf. Biogeosciences.

[8] Xu H., Song W., Warren A., Al-Rasheid K.A.S., Al-Farraj S.A., Gong J., Hu X. (2008). Planktonic protist communities in a semi-enclosed mariculture pond: Structural variation and correlation with environmental conditions. J. Mar. Biol. Assoc. UK.

[9] El-Tohamy W.S., Hopcroft R.R. (2024). Planktonic ciliate communities along an environmental gradient in the Nile Delta (Damietta region, Egypt). Sci. Rep..

[10] Zhang G., Yao T., Xie H., Kang S., Yi C., Han J., Chen X., Wang J., Yang K. (2020). Response of Tibetan Plateau lakes to climate change: Trends, patterns, and mechanisms. Earth-Sci. Rev..

[11] Wu G., Duan A., Liu Y., Mao J., Ren R., Bao Q., Chen D., Liu Y., Hu W., Zhang Y. (2015). Tibetan Plateau climate dynamics: Recent research progress and outlook. Natl. Sci. Rev..

[12] Zhang W., Liu L., Wu H., Zhang Y., Chen D. (2025). Snow droughts amplify compound climate extremes over the Tibetan Plateau. Commun. Earth Environ..

[13] Zhu L., Ju J., Qiao B., Liu C., Wang J., Yang R., Ma Q., Guo L., Pang S. (2025). Physical and biogeochemical responses of Tibetan Plateau lakes to climate change. Nat. Rev. Earth Environ..

[14] Barouillet C., Vasselon V., Keck F., Millet L., Etienne D., Galop D., Rius D., Domaizon I. (2022). Paleoreconstructions of ciliate communities reveal long-term ecological changes in temperate lakes. Sci. Rep..

[15] Weisse T. (2024). Thermal response of freshwater ciliates: Can they survive at elevated lake temperatures?. Freshw. Biol..

[16] Liu Y., An R., Wang C., Pan C., Ba S. (2023). Horizontal and Vertical Distributions of the Summer Phytoplankton Community in Basomtso Lake of Xizang, China. J. Hydroecol..

[17] Zhou D., Wang D., Ou M., Gesang Q., De J., Liu L., Guo X. (2024). Bacterial Community Composition and Characterization of Molecular Ecological Networks in the Water Column of the Basomtso Lake. Acta Hydrobiol. Sin..

[18] Zhou D., Wang D., Ge S., Ou M., Guo X., De J. (2025). Fungal Diversity, Community Structure and Prediction of Ecological Function in Basomtso Lake, Xizang. Biotechnol. Bull..

[19] An R., Pan C., Taba L., Yang X., Ba S. (2021). Vertical distribution characteristics of phytoplankton functional groups and their relationships with environmental factors in Lake Basomtso, Tibet, China. J. Lake Sci..

[20] Liu M., Zhu F., Zhu T., Li L., Wang L., Liu X., Zhu R., Liu F., Cen X., Hu F. (2025). Status of aquatic organisms resources and their environments in Xizang (2017–2021). J. Fish. China.

[21] Martin M. (2011). Cutadapt removes adapter sequences from high-throughput sequencing reads. EMBnet. J..

[22] Bokulich N.A., Kaehler B.D., Rideout J.R., Dillon M., Bolyen E., Knight R., Huttley G.A., Caporaso J.G. (2018). Optimizing taxonomic classification of marker-gene amplicon sequences with QIIME 2’s q2-feature-classifier plugin. Microbiome.

[23] Bolyen E., Rideout J.R., Dillon M.R., Bokulich N.A., Abnet C.C., Al-Ghalith G.A., Alexander H., Alm E.J., Arumugam M., Asnicar F. (2019). Reproducible, interactive, scalable and extensible microbiome data science using QIIME 2. Nat. Biotechnol..

[24] Callahan B.J., McMurdie P.J., Rosen M.J., Han A.W., Johnson A.J.A., Holmes S.P. (2016). DADA2: High-resolution sample inference from Illumina amplicon data. Nat. Methods.

[25] Guillou L., Bachar D., Audic S., Bass D., Berney C., Bittner L., Boutte C., Burgaud G., de Vargas C., Decelle J. (2013). The Protist Ribosomal Reference database (PR2): A catalog of unicellular eukaryote small sub-unit rRNA sequences with curated taxonomy. Nucleic Acids Res..

[26] Zhou T., Xu K., Zhao F., Liu W., Li L., Hua Z., Zhou X. (2023). itol.toolkit accelerates working with iTOL (Interactive Tree of Life) by an automated generation of annotation files. Bioinformatics.

[27] Letunic I., Bork P. (2021). Interactive Tree Of Life (iTOL) v5: An online tool for phylogenetic tree display and annotation. Nucleic Acids Res..

[28] Hijmans R.J., Williams E., Vennes C. (2017). Package ‘geosphere’. Spher. Trigonometry.

[29] R Core Team (2018). R: A Language and Environment for Statistical Computing. R Foundation for Statistical Computing. https://www.R-project.org/.

[30] Kembel S.W., Cowan P.D., Helmus M.R., Cornwell W.K., Morlon H., Ackerly D.D., Blomberg S.P., Webb C.O. (2010). Picante: R tools for integrating phylogenies and ecology. Bioinformatics.

[31] Isobe K., Bouskill N.J., Brodie E.L., Sudderth E.A., Martiny J.B.H. (2020). Phylogenetic conservation of soil bacterial responses to simulated global changes. Philos. Trans. R. Soc. B.

[32] Orme D., Freckleton R., Thomas G., Petzoldt T., Fritz S., Isaac N., Pearse W. (2011). Caper: Comparative Analyses of Phylogenetics and Evolution in R. https://CRAN.R-project.org/package=caper.

[33] Wang X., Wang Z., Liu W., Liu H., Zhang Q., Zeng J., Wang H., Zhu J., Li Y. (2023). Abundant and rare fungal taxa exhibit different patterns of phylogenetic niche conservatism and community assembly across a geographical and environmental gradient. Soil Biol. Biochem..

[34] Chen W., Ren K., Isabwe A., Chen H., Liu M., Yang J. (2019). Stochastic processes shape microeukaryotic community assembly in a subtropical river across wet and dry seasons. Microbiome.

[35] Stegen J.C., Lin X., Fredrickson J.K., Chen X., Kennedy D.W., Murray C.J., Rockhold M.L., Konopka A. (2013). Quantifying community assembly processes and identifying features that impose them. ISME J..

[36] Stone L., Roberts A. (1990). The checkerboard score and species distributions. Oecologia.

[37] Liaw A., Wiener M. (2002). Classification and regression by randomForest. R News.

[38] Clarke K.R., Ainsworth M. (1993). A method of linking multivariate community structure to environmental variables. Mar. Ecol. Prog. Ser..

[39] McMurdie P.J., Holmes S. (2013). phyloseq: An R package for reproducible interactive analysis and graphics of microbiome census data. PLoS ONE.

[40] Diniz-Filho J.A.F., Soares T.N., Lima J.S., Dobrovolski R., Landeiro V.L., de Campos Telles M.P., Rangel T.F., Bini L.M. (2013). Mantel test in population genetics. Genet. Mol. Biol..

[41] Baker M.E., King R.S. (2010). A new method for detecting and interpreting biodiversity and ecological community thresholds. Methods Ecol. Evol..

[42] Wang Z., Chi Y., Li T., Li L., Zhang Y., Liu Y., Li C., Li X., Song W. (2022). Biodiversity of freshwater ciliate (Protista, Ciliophora) in the Lake Weishan Wetland, China: The state of the art. Mar. Life Sci. Technol..

[43] Huang P.-P., Zhao F., Xu K.-D. (2020). Effects of sedimentation of DNA from overlying waters on the evaluation of ciliate molecular diversity in offshore sediments. Oceanol. Limnol. Sin..

[44] Majaneva M., Diserud O.H., Eagle S.H.C., Boström E., Hajibabaei M., Ekrem T. (2018). Environmental DNA filtration techniques affect recovered biodiversity. Sci. Rep..

[45] Sun P., Huang L., Xu D., Warren A., Huang B., Wang Y., Wang L., Xiao W., Kong J. (2019). Integrated space-time dataset reveals high diversity and distinct community structure of ciliates in mesopelagic waters of the northern South China Sea. Front. Microbiol..

[46] Novák J., Treitli S.C., Füssy Z., Záhonová K., Hamplová B., Hrdá Š., Hampl V. (2024). V9 hypervariable region metabarcoding primers for Euglenozoa and Metamonada. Environ. DNA.

[47] Huang Q., Li M., Li T., Zhu S., Wang Z., Pu B. (2024). Spatiotemporal distribution patterns of soil ciliate communities in the middle reaches of the Yarlung Zangbo River. Front. Environ. Sci..

[48] Tan Y., Huang L., Huang X., Zeng Z. (2010). The relationships between ciliate composition, abundance, and environmental factors in Sanya Bay coral reef waters. Acta Ecol. Sin..

[49] Zhu S., Huang Q., Li T., Li M., Pu B. (2024). Soil water content drives the spatiotemporal distribution and community assembly of soil ciliate in the Nianchu River Basin, Qinghai-Tibet Plateau, China. PLoS ONE.

[50] Lian K., Liu F., Li Y., Li R., Su Y., Wang X., Wang H., Chen Y., Zhang S., Liu J. (2023). Environmental gradients shape microbiome assembly and stability in the East China Sea. Environ. Res..

[51] Buonanno F., Ortenzi C. (2018). Predator-prey interactions in ciliated protists. Extremophilic Microbes and Metabolites—Diversity, Bioprospecting and Biotechnological Applications.

[52] Vďačný P., Foissner W. (2012). Monograph of the dileptids (Protista, Ciliophora, Rhynchostomatia). Denisia.

[53] Liu W., McManus G.B., Lin X., Huang H., Zhang W., Tan Y. (2021). Distribution patterns of ciliate diversity in the South China Sea. Front. Microbiol..

[54] Zhang J., Kong J.D., Shi J., Wang H. (2021). Phytoplankton competition for nutrients and light in a stratified lake: A mathematical model connecting epilimnion and hypolimnion. J. Nonlinear Sci..

[55] Yu Z., Yang J., Amalfitano S., Yu X., Liu L. (2014). Effects of water stratification and mixing on microbial community structure in a subtropical deep reservoir. Sci. Rep..

[56] Wu H., Bertilsson S., Li Y., Zhang W., Niu L., Cai W., Cong H., Zhang C. (2024). Influence of rapid vertical mixing on bacterial community assembly in stratified water columns. Environ. Res..

[57] Brenes-Guillén L., Vidaurre-Barahona D., Avilés-Vargas L., Castro-Gutiérrez V., Gómez-Ramírez E., González-Sánchez K., Mora-López M., Umaña-Villalobos G., Uribe-Lorío L., Hassard F. (2022). First insights into the prokaryotic community structure of Lake Cote, Costa Rica: Influence on nutrient cycling. Front. Microbiol..

[58] Wu K., Zhao W., Li M., Picazo F., Soininen J., Shen J., Zhu L., Cheng X., Wang J. (2020). Taxonomic dependency of beta diversity components in benthic communities of bacteria, diatoms and chironomids along a water-depth gradient. Sci. Total Environ..

[59] Wang D., Huang Y., Yang H. (2023). Differences of bacterial community co-occurrence network and assembly processes between sediment and water in lakes on the Qinghai-Tibet Plateau. J. Lake Sci..

[60] Wiens J.J., Graham C.H. (2005). Niche conservatism: Integrating evolution, ecology, and conservation biology. Annu. Rev. Ecol. Evol. Syst..

[61] Hsieh C., Gorczynski D., Bitariho R., Espinosa S., Johnson S.E., Lima M.G.M., Rovero F., Salvador J., Santos F., Sheil D. (2024). Evolutionary history and environmental variability structure contemporary tropical vertebrate communities. Glob. Ecol. Biogeogr..

[62] Martiny J.B.H., Jones S.E., Lennon J.T., Martiny A.C. (2015). Microbiomes in light of traits: A phylogenetic perspective. Science.

[63] Louca S., Polz M.F., Mazel F., Albright M.B.N., Huber J.A., O’Connor M.I., Ackermann M., Hahn A.S., Srivastava D.S., Crowe S.A. (2018). Function and functional redundancy in microbial systems. Nat. Ecol. Evol..

[64] Xie C., Gu J., Peng L., Wang C., Xu S., Hou S., Wang Z., Tang Y. (2025). Spatial heterogeneity dominates bacterial biogeography in the surface waters from the South China Sea by structuring environmental gradients. Microbiol. Spectr..

[65] Xie Z., Li W., Yang K., Wang X., Xiong S., Zhang X. (2024). Bacterial and archaeal communities in Erhai Lake sediments: Abundance and metabolic insight into a plateau lake at the edge of eutrophication. Microorganisms.

[66] Isobe K., Allison S.D., Khalili B., Martiny A.C., Martiny J.B.H. (2019). Phylogenetic conservation of bacterial responses to soil nitrogen addition across continents. Nat. Commun..

[67] Wang L.Y., Zhou H., Qin M., Shi M.W., Zheng X. (2024). Correlation of environmental factors with bacterial community structure and function of river water flowing through urban areas in upper reaches of Baiyangdian Lake. Asian J. Ecotoxicol..

[68] Wang J.B., Huang L., Ju J.T., Daut G., Ma Q.F., Zhu L.P., Haberzettl T., Baade J., Mausbacher R., Hamilton A. (2020). Seasonal stratification of a deep, high-altitude, dimictic lake: Nam Co, Tibetan Plateau. J. Hydrol..

[69] Leppäranta M. (2023). History and future of snow and sea ice in the Baltic Sea. Oxford Research Encyclopedia of Climate Science.

[70] Rimet F., Lemonnier C., Alric B. (2025). Stochasticity shapes microbial communities in high-altitude lakes, whereas species selection and homogenization dispersal are more important in lowland lakes: Case of benthic diatoms in alpine lakes. Ecol. Evol..

[71] Couto T.B.A., Messager M.L., Olden J.D. (2021). Safeguarding migratory fish via strategic planning of future small hydropower in Brazil. Nat. Sustain..

[72] Mouquet N., Loreau M. (2003). Community patterns in source-sink metacommunities. Am. Nat..

[73] Deutschmann I.M., Delage E., Giner C.R., Sebastián M., Poulain J., Arístegui J., Duarte C.M., Acinas S.G., Massana R., Gasol J.M. (2024). Disentangling microbial networks across pelagic zones in the tropical and subtropical global ocean. Nat. Commun..

[74] Tutasi P., Escribano R. (2020). Zooplankton diel vertical migration and downward C flux into the oxygen minimum zone in the highly productive upwelling region off northern Chile. Biogeosciences.

[75] Sieber M., Pita L., Weiland-Bräuer N., Dirksen P., Wang J., Mortzfeld B., Franzenburg S., Schmitz R.A., Baines J.F., Fraune S. (2019). Neutrality in the metaorganism. PLoS Biol..

[76] Dini-Andreote F., Stegen J.C., van Elsas J.D., Salles J.F. (2015). Disentangling mechanisms that mediate the balance between stochastic and deterministic processes in microbial succession. Proc. Natl. Acad. Sci. USA.

[77] Horton D.J., Theis K.R., Uzarski D.G., Learman D.R. (2019). Microbial community structure and microbial networks correspond to nutrient gradients within coastal wetlands of the Laurentian Great Lakes. FEMS Microbiol. Ecol..

[78] Liu C., Wu F., Jiang X., Hu Y., Shao K., Tang X., Li X. (2022). Salinity is a key determinant for the microeukaryotic community in lake ecosystems of the Inner Mongolia plateau, China. Front. Microbiol..

[79] Schulz H.E., Simoes A.L.A., Lobosco R.J. (2012). Hydrodynamics: Natural Water Bodies.

[80] Kraemer B.M., Anneville O., Chandra S., Dix M., Kuusisto E., Livingstone D.M., Rimmer A., Schladow S.G., Silow E., Sitoki L.M. (2015). Morphometry and average temperature affect lake stratification responses to climate change. Geophys. Res. Lett..

[81] Zhan Q., Stratmann C.N., van der Geest H.G., Veraart A.J., Brenzinger K., Lürling M., de Senerpont Domis L.N. (2021). Effectiveness of phosphorus control under extreme heatwaves: Implications for sediment nutrient releases and greenhouse gas emissions. Biogeochemistry.

[82] Wang C., Zhao Y., Du P., Li Y., Wang Z., Wang Y., Zhang W. (2022). Planktonic ciliate community structure and its distribution in the oxygen minimum zones in the Bay of Bengal (eastern Indian Ocean). J. Sea Res..

[83] Lyu M., Potter H., Collins C.O., Tamizi E., Zippel S., Thomson J. (2023). The impacts of gustiness on the evolution of surface gravity waves. Geophys. Res. Lett..

